# 

**Published:** 1892-09-10

**Authors:** 


### CONTENTS.

| | PAGE |
|---|---|
| **Not Out of Danger** | 385 |
| **Passing Topics.**—Friends in Need | 386 |
| **The Doctor's Armchair.**—Past and Present | 387 |
| **Women as Workers.**—I. Where Women Must Fail | 388 |
| **Medical History from the Earliest Times.** By E. T. Withington, M.A., M.B.Oxon. XXIII.—Roman Military Medicine | 389 |
| **The Brine Bath Treatment of Cholera** | 390 |
| **Annotations.**—Milk and Scarlet Fever—Medical Sickness and Annuity—The Fever Hospitals Full | 391 |
| **Notes and News** | 392 |
| **Scraps and Gleanings** | 393 |
| **The Practitioner's Mirror and Retrospect**— | |
| Medicine.—Diet in Enteric Fever, 394; Treatment of Acute Pleuritis | 395 |
| Therapeutics.—Ephedra Vulgaris | 396 |
| **The Story of the Insane from Year to Year.**—New South Wales Asylum | 396 |
| **The Institutional Workshop**— | |
| Practical Departments. I.—The Laundry | 397 |
| Hospital Construction.—Forres Leanchoil Hospital | 398 |
| **The Cure of Poverty.**—An Account of the North Kensington Friendly Workers among the Poor. By Mrs. C. Furley-Smith | 399 |
| **The Student of Medicine.**—Vacancies—Past Lists | |

### EXTRA SUPPLEMENT.—THE NURSING MIRROR.

| | |
|---|---|
| **En Passant.**—Midwives Registration—Crumpsall Infirmary, Manchester—Nurses Co-operation—Long Life for Nurses—Lady Students—The Adelaide Hospital; Dublin—Women and Fire Drill—Tall or Short?—A Seasonable Suggestion | clxix |
| **Lectures for Asylum Attendants.** By William Harding, M.B. I.—Introductory (*concluded*) | clxx |
| **Where to Go** | clxx |
| **How to Become a Medical Woman** | clxxi |
| **For Reading to the Sick.**—Unbelief | clxxii |
| **Presentations** | clxxiii |
| **Death in Our Ranks** | clxxiii |
| **Everybody's Opinion.**—Metropolitan Fever Hospitals—Cholera and Common Sense—Cholera Preparations at the Hospitals—Hospital Arrangements—An Old Hand—A Private Nurse's Report—General Hospital, Birmingham—Removal of the Brassey Home—Cholera Lectures to Nurses | clxxii |
| **Appointments** | clxxiii |
| **Notes and Queries** | clxxiii |
| **Hints to Nurses** | clxxiii |
| **Off Duty.** — "The Result of a Chill." I.—The Hermits of Dartmoor | clxxiv |
| **Wants and Workers** | clxxiv |

